# Synthesis and Evaluation of the Anticonvulsant Activities of 4-(2-(Alkylthio)benzo[*d*]oxazol-5-yl)-2,4-dihydro-3*H*-1,2,4-triazol-3-ones

**DOI:** 10.3390/molecules23040756

**Published:** 2018-03-25

**Authors:** Ming-Xia Song, Zhen-Yuan Wang, Shi-Hui He, Sheng-Wang Yu, Shi-Long Chen, Dong-Fu Guo, Wen-Hao Zhao, Xian-Qing Deng

**Affiliations:** 1 Medical College, Jinggangshan University, No. 28, Xueyuan Road, Ji’an 343009, Jiangxi, China; freexiaoxiao83@aliyun.com (M.-X.S.); 18770606620@163.com (S.-H.H.); ysw1779326748@163.com (S.-W.Y.); chensl@126.com (S.-L.C.); guoran147369@163.com (D.-F.G.); Zhaowh@126.com (W.-H.Z.); 2 Siping Institute of Food and Drug Control, No. 357, Nanhu Road, Sipin 136000, Jilin, China; sy6180343@sina.com

**Keywords:** anticonvulsant, maximal electroshock, pentylenetetrazole, benzoxazole, triazolone

## Abstract

In this study, a novel series of 4-(2-(alkylthio)benzo[*d*]oxazol-5-yl)-2,4-dihydro-3*H*-1,2,4-triazol-3-ones (**4a**–**m**) was designed and synthesized. The anticonvulsant activities of these compounds were evaluated by using the maximal electroshock seizure (MES) and subcutaneous pentylenetetrazole (scPTZ) seizure models in mice. The neurotoxicity of these compounds was evaluated using the rotarod neurotoxicity test. The majority of compounds showed anti-MES activities at 100 or 300 mg/kg. Compound **4g** was considered to be the most promising, based on its potency against MES- and PTZ-induced seizures with ED_50_ values of 23.7 and 18.9 mg/kg, respectively. The TD_50_ value of **4g** was 284.0 mg/kg, which resulted in a higher protective index (PI = TD_50_/ED_50_) value than that of carbamazepine and valproate. In an ELISA test, compound **4g** significantly increased the γ-aminobutyric acid (GABA) content in mouse brain. In addition, pretreatment with thiosemicarbazide (an inhibitor of the GABA synthesizing enzyme) significantly decreased the activity of **4g** in the MES model, which suggests that the mechanism through which compound **4g** elicits its anticonvulsive action is at least in part through increasing the GABA level in the brain.

## 1. Introduction

Epilepsy, a group of neurological disorders characterized by epileptic seizures, afflicts over 50 million people worldwide [1,2]. The cause of most cases of epilepsy is unknown. The accepted pathogenesis of epileptic seizures is the imbalance of excitatory and inhibitory neurotransmitters in the central nervous system, which leads to abnormal nerve cell activity and neuronal discharge, resulting in seizures [3]. Until now, anticonvulsants (also commonly known as antiepileptic drugs or antiseizure drugs), the “symptomatic” agents that suppress the symptoms of epilepsy (i.e., seizures), have been the main strategy for the treatment of epilepsy. However, the available anticonvulsants are only effective in reducing the severity and number of seizures in <70% of patients. Furthermore, the use of anticonvulsants is often associated with numerous undesirable side effects [4,5] and even life-threatening conditions [6]. Research to identify more effective and safer antiepileptic drugs is therefore an imperative yet challenging task for medicinal chemistry.

**Figure 1:**
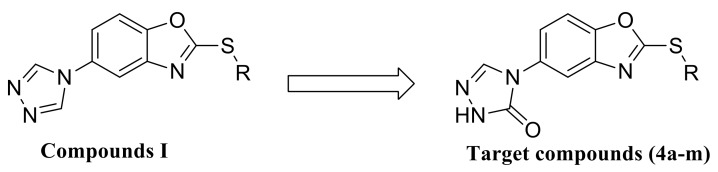
The leading compounds and target compounds (**4a**–**m**).

In our previous study, we reported the synthesis and anticonvulsant activity of 2-thioalkyl-5-(4*H*-1,2,4-triazol-4-yl)benzo[*d*]oxazoles (Figure 1, **I**) [7]. This series of compounds exhibited potent anticonvulsant activities. Among them, 2-(3-fluobenzyl)thio-5-(4*H*-1,2,4-triazol-4-yl)benzo[*d*]oxazole was the most potent with an ED_50_ value of 11.4 and 31.7 mg/kg in maximal electroshock seizure (MES) and subcutaneous pentylenetetrazole induced seizure (sc-PTZ) models, respectively. Preliminary research investigating the mechanism of action suggested that the GABAergic system may contribute to the anticonvulsive action of this compound. As a continuation of the above study, in the current study, a series of benzoxazole derivatives containing triazolone (**4a**–**m**) were designed and synthesized, which replaced the triazole in compounds of **I** with triazolone. This design is based on a hypothesis that the carbonyl group in the triazolone may increase the affinity to the receptor, and thus increase the anticonvulsant activity, which has already been confirmed in previous studies [8,9].

MES and scPTZ models, along with a toxicity screen (rota-rod in mice), were employed to screen the new anticonvulsants. These are the most widely used animal models in the discovery of new anticonvulsant drugs at the initial stage [10,11]. To further confirm the anticonvulsant activity of **4g**, it was also tested in 3-mercaptopropionic acid (3-MP), and bicuculline (BIC)-induced seizure models. In addition, studies investigating the involvement of the GABAergic system in the anticonvulsant mechanism of **4g** were also performed.

## 2. Results and Discussion

### 2.1. Chemistry

**Scheme 1:**
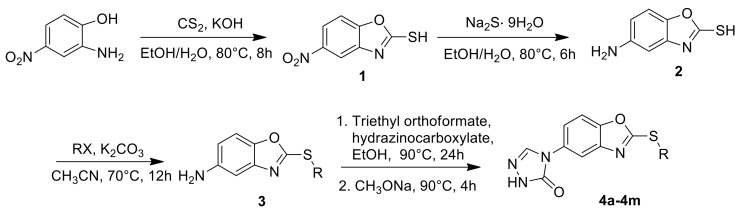
The synthesis route of target compounds (**4a**–**m**).

The target compounds were synthesized according to Scheme 1. The starting material, 2-amino-5-nitrophenol, was reacted with CS_2_ to obtain compound **1**, which was reduced further by Na_2_S·9H_2_O in ethanol to obtain compound **2** [12]. Alkylation of compound **2** with a variety of different alkylating agents produced compounds **3**, which were then treated with triethyl orthoformate and methyl hydrazine carboxylate in the presence of sodium methoxide to give rise to the target compounds **4a**–**m** [13]. The chemical structures of these compounds were characterized using ^1^H-NMR, ^13^C-NMR, and mass spectrum (MS) analysis techniques. A detailed overview of the physical and analytical data from these compounds has been provided in the Materials and Methods section.

### 2.2. Anticonvulsant Activity in MES and scPTZ Models and Neurotoxicity in the Rota-Rod Test

MES and scPTZ models, along with toxicity screen (rota-rod in mice), were employed to screen the new compounds [10,11]. Compounds **4a**–**m** were initially administered at doses of 30, 100 and 300 mg/kg. As shown in Table 1, compounds **4a** and **4e**–**i** displayed noticeable anticonvulsant activity at a dosage of 100 mg/kg in the MES model, with some compounds remaining active after 4 h. Compounds **4b, 4j** and **4k** displayed weak activity at a dosage of 300 mg/kg in the MES model. In the scPTZ model, compounds **4a**, **4h** and **4l** protected the mice from sc-PTZ induced seizure at a dosage of 300 mg/kg. Compounds **4e** and **4f** prevented seizures at a dose of 100 mg/kg in the scPTZ model at 0.5 h, and remained active after 4 h at the same dose. Compound **4g** was the most promising compound. Compound **4g** prevented seizures at a dose of 30 mg/kg in both models, though it was also the only compound that showed neurotoxicity at a dose of 300 mg/kg. In the toxicity screen, all compounds except **4g** showed no neurotoxicity at a dose of 300 mg/kg. Additionally, common side effects of anticonvulsant drugs, such as sedation, hypnosis, and anxiety (with features of bradykinesia or running), were not observed for any of the compounds at a dose of 100 mg/kg. However, at a higher dose of 300 mg/kg, compounds **4e**–**4f** caused sedation in the test animals to a certain extent.

**Figure 2:**
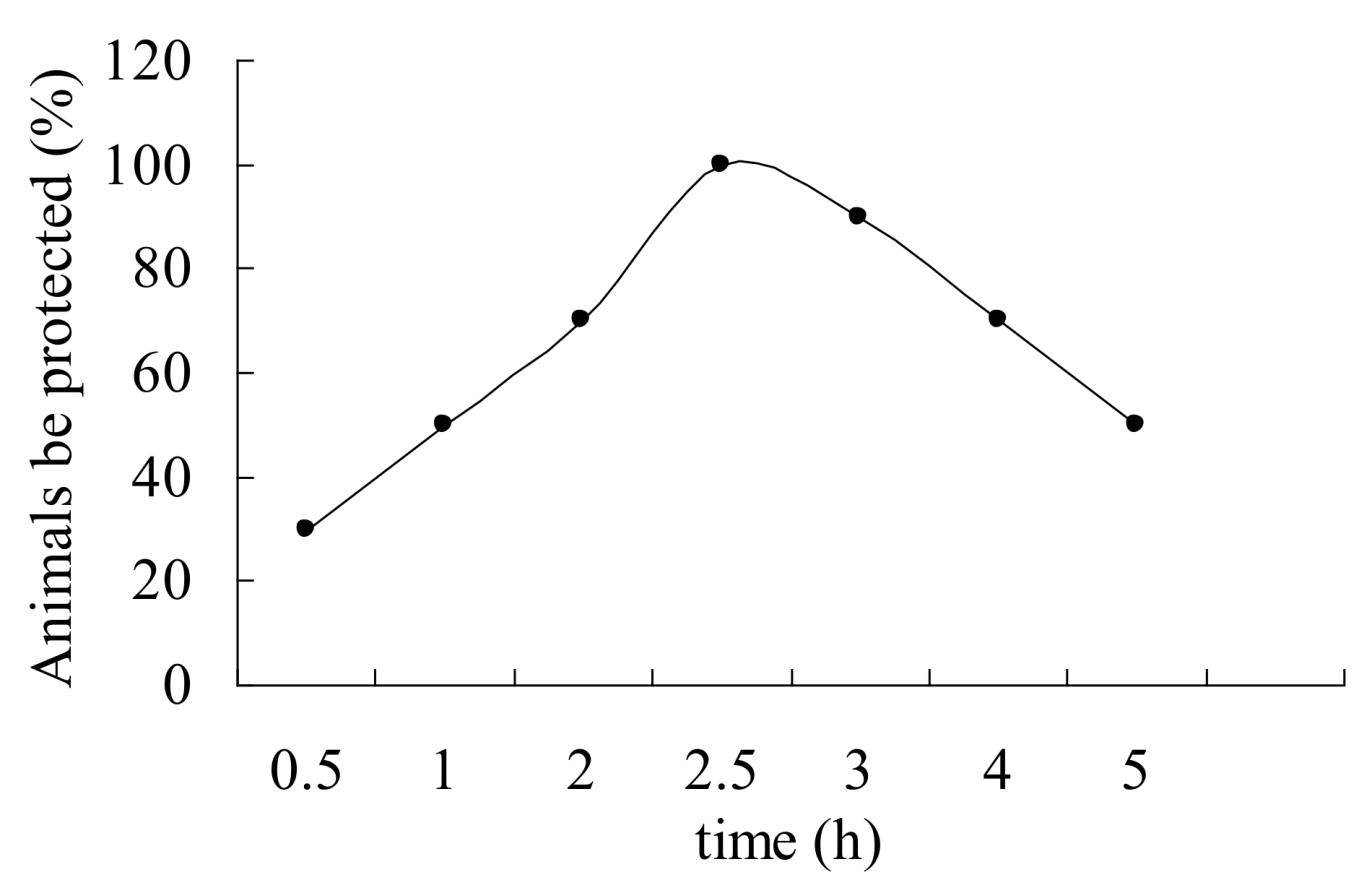
Time-course of compound **4g** (50 mg/kg) in the maximal electroshock seizure test (i.p.). The data illustrates % animal protected relative to animal tested (10 for each point) at various times after injection.

From the data in Table 1, it can be seen that some compounds, including **4g**, remained active until the 4 h time point. This indicates that these compounds possessed anticonvulsant activity with protective effects for a long duration. To evaluate the anticonvulsant activity of **4g** more accurately, the time to peak effect (TPE) was tested. Compound **4g** reached the TPE at 2.5 h after i.p. administration (Figure 2). Therefore, the 2.5 h time interval was chosen as the assessment time for compound **4g** in further quantification tests.

Based on the considerable anticonvulsant activity of **4g** that was demonstrated in the preliminary screens, further quantitative evaluation trials for the quantification of anticonvulsant activity (indicated by ED_50_) and neurotoxicity (indicated by TD_50_) in mice were performed. The ED_50_ of **4g** was measured as 23.7 mg/kg in the MES model (Table 2). Compound **4g** showed a higher activity than the positive drugs carbamazepine and valproate in the scPTZ model, with an ED_50_ of 18.9 mg/kg. The TD_50_ of **4g** was measured as 284.0 mg/kg, which resulted in PI values of 12.0 and 15.0 for the MES and scPTZ models, respectively. Compound **4g** showed superior performance to phenobarbital and valproate in terms of safety.

### 2.3. Anticonvulsant Activity Evaluation of Compound ***4g*** in Other Seizure Models

To further evaluate the anticonvulsive ability of **4g**, 3-mercaptopropionic acid (3-MP)- and bicuculline (BIC)-induced seizure tests were carried out. The 3-MP is a competitive inhibitor of glutamate decarboxylase (GAD) that can inhibit the synthesis of GABA, decreasing the level of this neurotransmitter in the brain [14]. 3-MP at a dose of 60 mg/kg induced convulsions in 100% of the mice (Table 3). Combined treatment with 3-MP and **4g** led to a significant decrease in tonic convulsions and death when compared to the results following administration of 3-MP alone (from 100% to 20%, *p* < 0.01; and from 60% to 30%, *p* < 0.05, for convulsions and death, respectively). BIC is a competitive GABA_A_ receptor antagonist that is known to cause seizures in mice [15]. BIC induced convulsions in 100% of the mice at a dose of 5.4 mg/kg. As shown in Table 4, carbamazepine inhibited tonic seizure and death significantly. Comparably, compound **4g** also inhibited tonic seizure and death significantly (from 100% to 0, *p* < 0.001; and from 100% to 30%, *p* < 0.01, for convulsions and death, respectively). The efficacy of compound **4g** in inhibiting tonic seizures and death induced by 3-MP and BIC, at a level comparable with carbamazepine, further confirmed the antiepileptic action of **4g**.

### 2.4. The Role of the GABAergic System in the Anticonvulsant Mechanism of ***4g***

**Figure 3:**
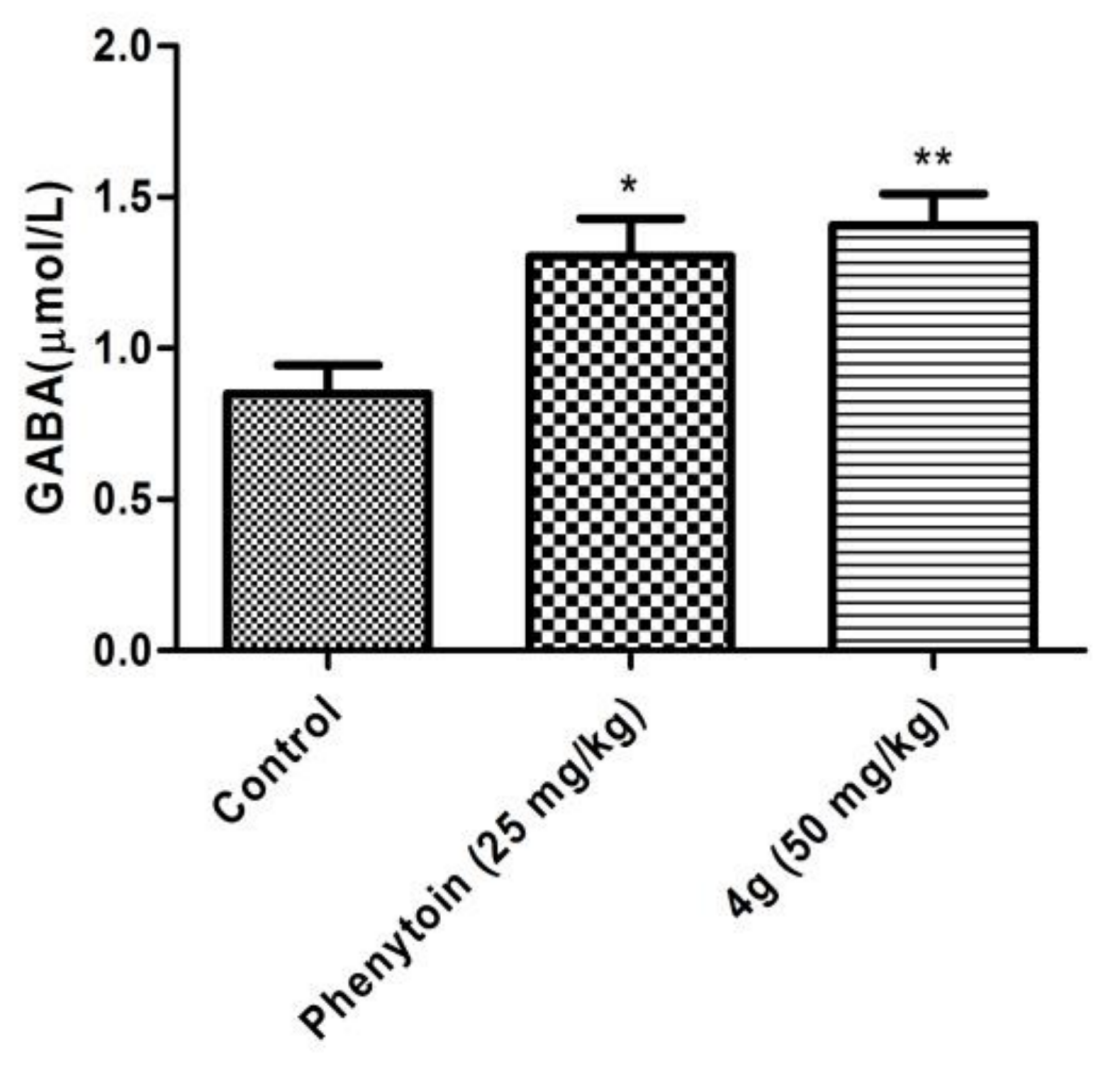
Effect of **4g** and phenytoin on whole brain GABA concentration in mice. Data are presented as mean ± SEM. of six animals. * *p* < 0.05, ** *p* < 0.01 vs. control (animals treated with saline).

In our previous study, the GABAergic system was involved in the anticonvulsive action of the compounds **I** [7]. Because of the structural similarity between the target compounds and the previous compounds, there is reason to believe that the mechanism through which these compounds produce an anticonvulsive action also involves the GABAergic system. An ELISA assay was performed to investigate the effects of **4g** on the GABA content in the mouse brain. As shown in Figure 3, compound **4g** and phenytoin significantly increased the GABA content in the mouse brain when compared to the results from the control group (*p* < 0.01 and *p* < 0.01, following treatment with **4g** and phenytoin respectively).

**Figure 4:**
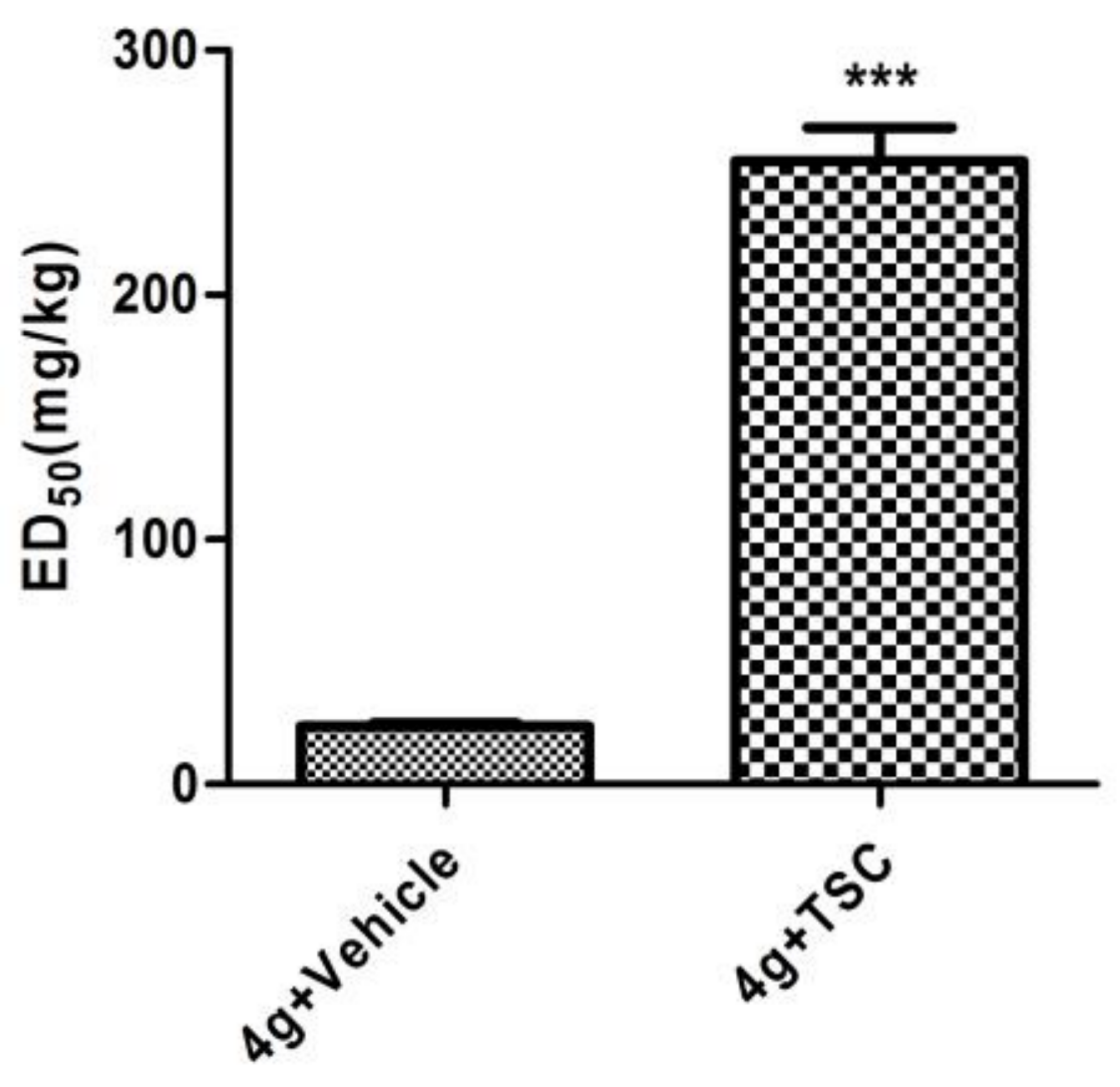
Effect of the thiosemicarbazide (TSC) on the anticonvulsive action of **4g** against maximal electroshock-induced seizures in mice. All data are presented as median effective dose (ED_50_) ± SEM. All drugs were administered i.p. in a single injection, 30 min before maximal electroshock-induced seizures; *** *p* < 0.001 vs. control group (pretreatment with saline).

To further confirm the anticonvulsant mechanism of **4g**, the effects of thiosemicarbazide (TSC) on the anticonvulsive action of **4g** were determined. As shown in Figure 4, pretreatment with TSC (25 mg/kg/day for 3 days) significantly decreased the seizure-preventing action of **4g** in the MES model. TSC is a competitive inhibitor of the GABA synthesis enzyme, glutamate decarboxylase (GAD), and it inhibits the synthesis of GABA, resulting in a decrease in the GABA level in the brain [16]. The above results further confirmed that the mechanism through which compound **4g** produces its anticonvulsive action is at least in part through increasing the GABA level in the brain.

## 3. Materials and Methods

### 3.1. Instruments and Reagents

All of the reagents and solvents were purchased from Aladdin (Shanghai, China) or Sinopharm Chemical Reagent Co. Ltd. (Shanghai, China), and were used as received. Melting points were determined in open capillary tubes and are uncorrected. Reaction courses were monitored by thin-layer chromatography on silica gel-precoated F_254_ plates (Merck, Darmstadt, Germany). Developed plates were examined with UV lamps (254 nm). Nuclear magnetic resonance spectroscopy was performed on an AV-300 spectrometer (Bruker, Zurich, Switzerland) operating at 300 MHz for ^1^H and 75 MHz for ^13^C and using DMSO-*d*_6_ as solvent and tetramethylsilane as the internal standard. An MALDI-TOF/TOF mass spectrometer (Bruker Daltonik, Bremen, Germany) was used to measure the high resolution mass spectroscopy.

### 3.2. Synthesis Method and Spectral Data

#### 3.2.1. General Procedure for the Preparation of Intermediates **1**, **2** and **3**

Intermediates **1**, **2** and **3** were synthesized according to the literature [7].

#### 3.2.2. General Procedure for the Preparation of Target Compounds **4a**–**m**

Compounds **3** (10 mmol), methyl hydrazinocarboxylate (50 mmol), and triethyl orthoformate (50 mmol) were placed into a round-bottomed flask containing 30 mL anhydrous alcohol and refluxed for 24 h. After cooling, sodium methylate (50 mmol) was added and refluxed. Following completion of the reaction (the mixture turning pink is a sign of completion), the mixture was cooled down to room temperature (20 °C) and diluted in 120 mL water and neutralized with HCl solution to pH 5–6. The precipitate that formed was filtered and washed with water, which was then purified by recrystallization with ethanol to obtain compounds **4a**–**m**.

#### 3.2.3. Spectral Data

*4-(2-(Propylthio)benzo[d]oxazol-5-yl)-2H-1,2,4-triazol-3(4H)-one* (**4a**). White solid; m.p. 202 °C, yield 31%. ^1^H-NMR (DMSO-*d*_6_, 300 MHz): *δ* 1.01 (t, 3H, *J* = 7.3 Hz, CH_2_CH_3_), 1.78–1.86 (m, 2H, CH_2_CH_3_), 3.32 (t, 2H, *J* = 7.1 Hz, SCH_2_), 7.63 (d, 1H, *J* = 7.4 Hz, Ph-H), 7.75 (d, 1H, *J* = 7.4 Hz, Ph-H), 7.94 (s, 1H, Ph-H), 8.38 (s, 1H, CH=N), 11.99 (s, 1H, CO-NH). ^13^C-NMR (DMSO-*d*_6_, 75 MHz): *δ* 166.8, 153.5, 150.1, 142.2, 137.1, 131.3, 118.6, 112.3, 110.9, 34.1, 22.8, 13.4. IR (KBr) cm^−1^: 3208 (N-H), 1703 (C=O). ESI-HRMS calcd. for C_12_H_13_N_4_O_2_S^+^ ([M + H]^+^): 277.0754; found: 277.0761.

*4-(2-(Pentylthio)benzo[d]oxazol-5-yl)-2H-1,2,4-triazol-3(4H)-one* (**4b**). White solid; m.p. 206–207 °C, yield 63%. ^1^H-NMR (DMSO-*d*_6_, 300 MHz): *δ* 0.87 (t, 3H, *J* = 6.8 Hz, CH_2_CH_3_), 1.31–1.37 (m, 4H, (CH_2_)_2_CH_3_), 1.75–1.80 (m, 2H, SCH_2_CH_2_), 3.32 (t, 2H, *J* = 7.0 Hz, SCH_2_), 7.62 (d, 1H, *J* = 8.5 Hz, Ph-H), 7.75 (d, 1H, *J* = 8.5 Hz, Ph-H), 7.94 (s, 1H, Ph-H), 8.38 (s, 1H, CH=N), 12.00 (s, 1H, CO-NH). ^13^C-NMR (DMSO-*d*_6_, 75 MHz): *δ* 166.8, 153.5, 150.1, 142.2, 137.1, 131.3, 118.6, 112.3, 110.9, 32.2, 30.6, 29.0, 22.1, 14.3. IR (KBr) cm^−1^: 3207 (N-H), 1702 (C=O). ESI-HRMS calcd for C_14_H_17_N_4_O_2_S^+^ ([M + H]^+^): 305.1067; found: 305.1076.

*4-(2-(Heptylthio)benzo[d]oxazol-5-yl)-2H-1,2,4-triazol-3(4H)-one* (**4c**). White solid; m.p. 201–202 °C, yield 53%. ^1^H-NMR (DMSO-*d*_6_, 300 MHz): *δ* 0.85 (t, 3H, *J* = 6.4 Hz, CH_2_CH_3_), 1.26–1.41 (m, 8H, (CH_2_)_4_CH_3_), 1.73–1.89 (m, 2H, SCH_2_CH_2_), 3.33 (t, 2H, *J* = 7.2 Hz, SCH_2_), 7.63 (dd, 1H, *J*_1_ = 8.7 Hz, *J*_2_ = 1.8 Hz, Ph-H), 7.74 (d, 1H, *J* = 8.7 Hz, Ph-H), 7.94 (d, 1H, *J* = 1.8 Hz, Ph-H), 8.38 (s, 1H, CH=N), 11.96 (s, 1H, CO-NH). ^13^C-NMR (DMSO-*d*_6_, 75 MHz): *δ* 166.8, 153.5, 150.0, 142.2, 137.1, 131.3, 118.4, 112.2, 110.9, 32.3, 31.6, 29.3, 28.6, 28.4, 22.5, 14.4. IR (KBr) cm^−1^: 3206 (N-H), 1701 (C=O). ESI-HRMS calcd for C_16_H_21_N_4_O_2_S^+^ ([M + H]^+^): 333.1380; found: 333.1373.

*4-(2-(Benzylthio)benzo[d]oxazol-5-yl)-2H-1,2,4-triazol-3(4H)-one* (**4d**). White solid; m.p. 185–186 °C, yield 28%. ^1^H-NMR (DMSO-*d*_6_, 300 MHz): *δ* 4.63 (s, 2H, SCH_2_), 7.28–7.52 (m, 5H, benzyl-H), 7.66 (dd, 1H, *J*_1_ = 8.5 Hz, *J*_2_ = 1.6 Hz, Ph-H), 7.76 (d, 1H, *J* = 8.5 Hz, Ph-H), 8.05 (d, 1H, *J* = 1.6 Hz, Ph-H), 8.42 (s, 1H, CH=N), 12.07 (s, 1H, CO-NH). ^13^C-NMR (DMSO-*d*_6_, 75 MHz): *δ* 165.5, 153.4, 151.6, 140.2, 137.0, 136.9, 130.9, 129.5, 129.1, 128.2, 119.0, 118.9, 104.8, 36.1. IR (KBr) cm^−1^: 3192 (N-H), 1703 (C=O). ESI-HRMS calcd for C_16_H_13_N_4_O_2_S^+^ ([M + H]^+^): 325.0754; found: 325.0746.

*4-(2-(2-Fluorobenzylthio)benzo[d]oxazol-5-yl)-2H-1,2,4-triazol-3(4H)-one* (**4e**). White solid; m.p. 198–199 °C, yield 41%. ^1^H-NMR (DMSO-*d*_6_, 300 MHz): *δ* 4.67 (s, 2H, SCH_2_), 7.16–7.38 (m, 3H, benzyl-H), 7.46–7.61 (m, 1H, benzyl-H), 7.66 (dd, 1H, *J*_1_ = 8.6 Hz, *J*_2_ = 1.6 Hz, Ph-H), 7.78 (d, 1H, *J* = 8.6 Hz, Ph-H), 7.98 (d, 1H, *J* = 1.6 Hz, Ph-H), 8.40 (s, 1H, CH=N), 12.00 (s, 1H, CO-NH). ^13^C-NMR (DMSO-*d*_6_, 75 MHz): *δ* 165.6, 160.9 (d, ^1^*J*_c-f_ = 243.8 Hz), 153.6, 150.2, 142.1, 137.1, 131.8, 131.6 (d, ^2^*J*_c-f_ = 29.3 Hz), 130.6 (d, ^3^*J*_c-f_ = 8.0 Hz), 125.1 (d, ^4^*J*_c-f_ = 3.4 Hz), 123.9 (d, ^3^*J*_c-f_ = 14.1 Hz), 119.0, 116.0 (d, ^2^*J*_c-f_ = 20.1 Hz), 112.5, 111.1, 30.0. IR (KBr) cm^−1^: 3190 (N-H), 1705 (C=O). ESI-HRMS calcd for C_16_H_12_FN_4_O_2_S^+^ ([M + H]^+^): 343.0660; found: 343.0652.

*4-(2-(3-Fluorobenzylthio)benzo[d]oxazol-5-yl)-2H-1,2,4-triazol-3(4H)-one* (**4f**). White solid; m.p. 220–222 °C, yield 46%. ^1^H-NMR (DMSO-*d*_6_, 300 MHz): *δ* 4.66 (s, 2H, SCH_2_), 7.16–7.64 (m, 4H, benzyl-H), 7.67 (dd, 1H, *J*_1_ = 8.6 Hz, *J*_2_ = 1.7 Hz, Ph-H), 7.78 (d, 1H, *J* = 8.6 Hz, Ph-H), 8.07 (d, 1H, *J* = 1.7 Hz, Ph-H), 8.43 (s, 1H, CH=N), 11.98 (s, 1H, CO-NH). ^13^C-NMR (DMSO-*d*_6_, 75 MHz): *δ* 164.9, 160.9 (d, ^1^*J*_c-f_ = 244.5 Hz), 153.5, 151.6, 140.1, 137.0, 131.8 (d, ^3^*J*_c-f_ = 3.5 Hz), 130.9 (d, ^2^*J*_c-f_ = 20.5 Hz), 130.6, 125.1 (d, ^4^*J*_c-f_ = 3.4 Hz), 123.8 (d, ^3^*J*_c-f_ = 14.5 Hz), 119.1, 119.0, 116.0 (d, ^2^*J*_c-f_ = 20.8 Hz), 104.8, 29.9. IR (KBr) cm^−1^: 3189 (N-H), 1709 (C=O). ESI-HRMS calcd for C_16_H_12_FN_4_O_2_S^+^ ([M + H]^+^): 343.0660; found: 343.0656.

*4-(2-(4-Fluorobenzylthio)benzo[d]oxazol-5-yl)-2H-1,2,4-triazol-3(4H)-one* (**4g**). White solid; m.p. 218–219 °C, yield 43%. ^1^H-NMR (DMSO-*d*_6_, 300 MHz): *δ* 4.63 (s, 2H, SCH_2_), 7.15–7.21 (m, 2H, benzyl-H), 7.54–7.59 (m, 2H, benzyl-H), 7.67 (dd, 1H, *J*_1_ = 8.6 Hz, *J*_2_ = 2.0 Hz, Ph-H), 7.77 (d, 1H, *J* = 8.6 Hz, Ph-H), 8.05 (d, 1H, *J* = 2.0 Hz, Ph-H), 8.42 (s, 1H, CH=N), 11.99 (s, 1H, CO-NH). ^13^C-NMR (DMSO-*d*_6_, 75 MHz): *δ* 165.4, 162.1 (d, ^1^*J*_c-f_ = 242.7 Hz), 153.6, 151.6, 140.1, 136.9, 133.3 (d, ^4^*J*_c-f_ = 3.0 Hz), 131.6 (d, ^3^*J*_c-f_ = 8.3 Hz), 131.0, 119.0, 118.9, 116.1 (d, ^2^*J*_c-f_ = 21.4 Hz), 104.7, 35.2. IR (KBr) cm^−1^: 3186 (N-H), 1710 (C=O). ESI-HRMS calcd for C_16_H_12_FN_4_O_2_S^+^ ([M + H]^+^): 343.0660; found: 343.0650.

*4-(2-(2-Chlorobenzylthio)benzo[d]oxazol-5-yl)-2H-1,2,4-triazol-3(4H)-one* (**4h**). White solid; m.p. 200–201 °C, yield 40%. ^1^H-NMR (DMSO-*d*_6_, 300 MHz): *δ* 4.63 (s, 2H, SCH_2_), 7.36–7.60 (m, 4H, benzyl-H), 7.66 (dd, 1H, *J*_1_ = 8.5 Hz, *J*_2_ = 1.7 Hz, Ph-H), 7.76 (d, 1H, *J* = 8.5 Hz, Ph-H), 8.05 (d, 1H, *J* = 1.7 Hz, Ph-H), 8.41 (s, 1H, CH=N), 12.05 (s, 1H, CO-NH). ^13^C-NMR (DMSO-*d*_6_, 75 MHz): *δ* 165.3, 153.4, 151.6, 140.1, 139.7, 136.9, 133.5, 133.4, 131.0, 130.9, 129.3, 128.2, 119.1, 118.9, 104.8, 35.3. IR (KBr) cm^−1^: 3194 (N-H), 1711 (C=O). ESI-HRMS calcd for C_16_H_12_ClN_4_O_2_S^+^ ([M + H]^+^): 359.0364; found: 359.0372.

*4-(2-(3-Chlorobenzylthio)benzo[d]oxazol-5-yl)-2H-1,2,4-triazol-3(4H)-one* (**4i**). White solid; m.p. 188–190 °C, yield 34%. ^1^H-NMR (DMSO-*d*_6_, 300 MHz): *δ* 4.64 (s, 2H, SCH_2_), 7.35–7.50 (m, 3H, benzyl-H), 7.60 (s, 1H, benzyl-H), 7.64 (dd, 1H, *J*_1_ = 8.8 Hz, *J*_2_ = 1.7 Hz, Ph-H), 7.77 (d, 1H, *J* = 8.8 Hz, Ph-H), 7.98 (d, 1H, *J* = 1.7 Hz, Ph-H), 8.39 (s, 1H, CH=N), 11.99 (s, 1H, CO-NH). ^13^C-NMR (DMSO-*d*_6_, 75 MHz): *δ* 165.9, 153.5, 150.2, 142.1, 139.8, 137.1, 133.5, 131.4, 130.9, 129.3, 128.2, 128.0, 118.9, 112.4, 111.1, 35.3. IR (KBr) cm^−1^: 3197 (N-H), 1712 (C=O). ESI-HRMS calcd for C_16_H_12_ClN_4_O_2_S^+^ ([M + H]^+^): 359.0364; found: 359.0356.

*4-(2-(4-Chlorobenzylthio)benzo[d]oxazol-5-yl)-2H-1,2,4-triazol-3(4H)-one* (**4j**). White solid; m.p. 225–227 °C, yield 54%. ^1^H-NMR (DMSO-*d*_6_, 300 MHz): *δ* 4.63 (s, 2H, SCH_2_), 7.41 (d, 2H, *J* = 8.4 Hz, benzyl-H), 7.55 (d, 2H, *J* = 8.4 Hz, benzyl-H), 7.67 (dd, 1H, *J*_1_ = 8.6 Hz, *J*_2_ = 1.9 Hz, Ph-H), 7.76 (d, 1H, *J* = 8.6 Hz, Ph-H), 8.05 (d, 1H, *J* = 1.9 Hz, Ph-H), 8.41 (s, 1H, CH=N), 12.02 (s, 1H, CO-NH). ^13^C-NMR (DMSO-*d*_6_, 75 MHz): *δ* 165.4, 153.5, 151.6, 140.2, 137.0, 136.3, 132.9, 131.4, 130.9, 129.1, 119.1, 119.0, 104.8, 35.3. IR (KBr) cm^−1^: 3195 (N-H), 1711 (C=O). ESI-HRMS calcd for C_16_H_12_ClN_4_O_2_S^+^ ([M + H]^+^): 359.0364; found: 359.0361.

*4-(2-(2,4-Dichlorobenzylthio)benzo[d]oxazol-5-yl)-2H-1,2,4-triazol-3(4H)-one* (**4k**). White solid; m.p. 171–172 °C, yield 45%. ^1^H-NMR (DMSO-*d*_6_, 300 MHz): *δ* 4.69 (s, 2H, SCH_2_), 7.41 (dd, 1H, *J*_1_ = 8.6 Hz, *J*_2_ = 1.8 Hz, Ph-H), 7.65–7.72 (m, 3H, benzyl-H), 7.76 (d, 1H, *J* = 8.6 Hz, Ph-H), 8.05 (d, 1H, *J* = 1.8 Hz, Ph-H), 8.42 (s, 1H, CH=N), 12.06 (s, 1H, CO-NH). ^13^C-NMR (DMSO-*d*_6_, 75 MHz): *δ* 164.8, 153.4, 151.6, 140.0, 136.9, 134.8, 134.0, 133.5, 133.1, 131.1, 129.6, 128.1, 120.5, 119.0, 104.7, 33.8. IR (KBr) cm^−1^: 3208 (N-H), 1707 (C=O). ESI-HRMS calcd for C_16_H_11_C_l2_N_4_O_2_S^+^ ([M + H]^+^): 392.9974; found: 392.9986.

*4-(2-(4-Methylbenzylthio)benzo[d]oxazol-5-yl)-2H-1,2,4-triazol-3(4H)-one* (**4l**). White solid; m.p. 230–231 °C, yield 55%. ^1^H-NMR (DMSO-*d*_6_, 300 MHz): *δ* 2.27 (s, 3H, Ar-CH_3_), 4.59 (s, 2H, SCH_2_), 7.15 (d, 2H, *J* = 7.4 Hz, benzyl-H), 7.39 (d, 2H, *J* = 7.4 Hz, benzyl-H), 7.64 (d, 1H, *J* = 8.6 Hz, Ph-H), 7.76 (d, 1H, *J* = 8.6 Hz, Ph-H), 7.97 (s, 1H, Ph-H), 8.39 (s, 1H, CH=N), 11.99 (s, 1H, CO-NH). ^13^C-NMR (DMSO-*d*_6_, 75 MHz): *δ* 166.2, 153.6, 150.1, 142.2, 137.5, 137.1, 133.8, 131.4, 129.6, 129.4, 118.8, 112.4, 111.0, 35.9, 21.2. IR (KBr) cm^−1^: 3204 (N-H), 1713 (C=O). ESI-HRMS calcd for C_17_H_15_N_4_O_2_S^+^ ([M + H]^+^): 339.0910; found: 339.0909.

*4-(2-(4-Methoxybenzylthio)benzo[d]oxazol-5-yl)-2H-1,2,4-triazol-3(4H)-one* (**4m**). White solid; m.p. 227–228 °C, yield 57%. ^1^H-NMR (DMSO-*d*_6_, 300 MHz): *δ* 3.72 (s, 3H, Ar-OCH_3_), 4.58 (s, 2H, SCH_2_), 6.90 (d, 2H, *J* = 8.4 Hz, benzyl-H), 7.43 (d, 2H, *J* = 8.4 Hz, benzyl-H), 7.63 (d, 1H, *J* = 8.7 Hz, Ph-H), 7.65 (d, 1H, *J* = 8.7 Hz, Ph-H), 7.97 (s, 1H, Ph-H), 8.40 (s, 1H, CH=N), 12.02 (s, 1H, CO-NH). ^13^C-NMR (DMSO-*d*_6_, 75 MHz): *δ* 166.2, 159.3, 153.6, 150.1, 142.2, 137.1, 131.3, 130.8, 128.6, 118.8, 114.5, 112.4, 111.0, 55.5, 35.7. IR (KBr) cm^−1^: 3202 (N-H), 1714 (C=O). ESI-HRMS calcd for C_17_H_15_N_4_O_3_S^+^ ([M + H]^+^): 355.0859; found: 355.0867.

### 3.3. Pharmacology

#### 3.3.1. Animals and Experimental Conditions

Experiments were carried out on KunMing mice that weighed 18–24 g. The mice were housed collectively in polycarbonate cages, in groups of ten, where they were maintained under a 12 h light/dark cycle in a temperature-controlled (25 ± 2 °C) laboratory with free access to food and water. Each animal was used only once. Procedures involving animals and their care were conducted in accordance with the Guide for the Care and Use of Laboratory Animals, 8th Edition, National Academies Press, Washington, DC, USA. Local ethical committee approval was also obtained (No. 20170701). Additionally, all efforts were made to minimize animal suffering and to use only the number of animals necessary to produce reliable scientific data. Electro-convulsions were produced by an electric stimulation generator (JTC-1, Chengdu, China). The following drugs were used in this study: pentylenetetrazole, 3-mercaptopropionic acid, thiosemicarbazide, and bicuculline (these drugs were obtained from Aladdin Industrial Inc., Shanghai, China), valproate sodium, carbamazepine, phenytoin (these drugs were obtained from melongpharma, Dalian, China).

#### 3.3.2. The Maximal Electroshock (MES) Test

Seizures were elicited in mice with ear stimulation using a 0.2 s 60-Hz 50-mA alternating current. Protection against the spread of MES-induced seizures was defined as the abolition of the hind limb tonic extension spasm [17,18].

#### 3.3.3. Subcutaneous Pentylenetetrazole Induced Seizures Test (scPTZ)

Pentylenetetrazole was dissolved in 0.9% saline to allow subcutaneous injections to mice at a dose of 85 mg/kg. The failure to observe even a threshold seizure (a single episode of clonic spasms of at least 5 s duration) is defined as protection [19].

#### 3.3.4. Neurotoxicity Screening

The neurotoxicity of the compounds was measured in mice using the rotarod test. The mice were trained to stay on an accelerating rotarod, with a diameter of 3.2 cm, that rotates at 10 rpm. Trained animals were given an intraperitoneal injection of the test compounds. Neurotoxicity was indicated by the inability of the animal to maintain equilibrium on the rod for at least 1 min in each of the trials [17,18].

#### 3.3.5. Quantification Trials

Quantitative anticonvulsant activity was expressed in terms of the median effective dose (ED_50_), that is, the dose of drug that is required to produce the biological responses in 50% of animals. Quantitative neurotoxicity was expressed as the median toxic dose (TD_50_). For determination of the ED_50_ and TD_50_, groups of ten mice were given a range of doses of the tested compounds until at least three points were established in the range of 10–90% seizure protection or neurotoxicity. From these data, the respective ED_50_ and TD_50_ values, and the 95% confidence intervals were calculated using the Graphpad prism 5. The PI values were calculated by dividing the TD_50_ by the ED_50_ [20].

#### 3.3.6. 3-Mercaptopropionic Acid-Induced Seizures Test

In the 3-Mercaptopropionic acid induced seizures test, the vehicle, carbamazepine (30 mg/kg) and **4g** (30 mg/kg) were administered, via i.p. injection, to groups of ten mice 30 min before the injection of 3-MP (60 mg/kg). The mice were placed in individual cages and observed for 60 min. The numbers of clonic seizures (categorized as behavior ranging from exaggerated twitches of the limbs to violent shaking or vibrating of the stiffened extremities) and tonic seizures (categorized as the extremities pulling towards the body or rigidly push away from it, usually with maximal extension of the hind leg), as well as the number of deaths were noted [14].

#### 3.3.7. Bicuculline-Induced Seizures Test

In the bicuculline induced seizures test, the vehicle, carbamazepine (30 mg/kg) and **4g** (30 mg/kg) were administered, via i.p. injection, to groups of ten mice 30 min before the injection of bicuculline (5.4 mg/kg). The mice were placed in individual cages and observed for 60 min. The numbers of clonic seizures (categorized as behavior ranging from exaggerated twitches of the limbs to violent shaking or vibrating of the stiffened extremities) and tonic seizures (categorized as the extremities pulling towards the body or rigidly push away from it, usually with maximal extension of the hind leg), as well as the number of deaths were noted [15].

#### 3.3.8. Determination of Brain GABA Concentrations by ELISA

Mice were randomly divided into three groups. The vehicle, phenytoin (25 mg/kg), and **4g** (50 mg/kg) were each administered via i.p. injection, to groups of ten mice. After 30 min, the mice were decapitated and the brains were rapidly removed, washed with cooled saline, and homogenized in 6 volumes (g/mL) of saline. The homogenate was centrifuged at 5000× *g* for 10 min at 4 °C. The GABA content of the supernatant was determined using enzyme-linked immunosorbent assay (ELISA) kits (Biolegend, San Diego, CA, USA) according to the manufacturer’s instructions. The GABA content of the brain was expressed as μmol/L [21].

#### 3.3.9. Test for the Effects of Thiosemicarbazide on the Anti-MES Action of **4g**

Pretreatment with TSC will significantly decrease the GABA levels in the brain of mice. In this study, thirty mice were pretreated with TSC at a dose of 25 mg/kg/day for three days. On the last day, the treated mice were used for the determination of the ED_50_ value of **4g** using the MES test. The ED_50_ value of **4g** in mice that were pretreated with TSC were obtained and compared to the previous ED_50_ value of **4g** achieved using the MES test [22].

#### 3.3.10. Statistical Analysis

In order to compare the ED_50_ values, the standard error of the mean (SEM) values were transformed from 95% confidence limits, and the ED_50_ values with the SEM were compared using one-way analysis of variance (ANOVA) followed by Dunnet’s test. The number of seizures and lethality from the chemical-induced seizure models were compared using the Fisher’s exact test. Differences among values were considered to be statistically significant if *P* ≤ 0.05. All statistical tests were performed using the commercially available GraphPad Prism version 5.0 for Windows (GraphPad Software Inc., La Jolla, CA, USA).

## 4. Conclusions

In summary, a series of benzoxazole derivatives containing triazolone (**4a-m**) were designed and synthesized. The anticonvulsant effects of these compounds were investigated using several kinds of seizure models. Compound **4g** was considered to be the most promising compound based on its potency against MES- and PTZ-induced seizures. The ED_50_ values of **4g** in these models were 23.7 and 18.9 mg/kg, respectively. The seizure-preventing action of **4g** in the 3-MP- and BIC-induced seizure models further confirmed its anticonvulsant activity. The protective activity of **4g** against BIC-induced seizures also suggested that the anticonvulsant activity of 4g involves the GABAergic system. Furthermore, experiments investigating the anticonvulsant mechanism of **4g** suggested that compound **4g** can increase GABA levels in the mouse brain and that this compound elicits its anticonvulsive action at least in part by increasing the GABA levels in the brain. A comparison of the previously synthesized compounds (**I**) with these triazolone compounds, with regard to their anticonvulsant activity, showed that there is no significant difference between them. Although the expectation of obtaining improved anticonvulsant activity using the triazolone moiety was not reached, compound **4g** exhibited a better anti-PTZ activity than the representative compound obtained in the previous study (18.9 mg/kg vs. 31.7 mg/kg).

## Figures and Tables

**Table 1:**
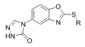
Anticonvulsant activity and neurotoxicity of compounds **4a**–**m** administered intraperitoneally to mice.

Compds.	R	Intraperitioneal Injection in Mice ^a^					
		MES Screening ^b^		scPTZ Screening ^c^		NT Screening ^d^	
		0.5 h	4 h	0.5 h	4 h	0.5 h	4 h
4a	C_3_H_7_	100	300	300	-	-	-
4b	C_5_H_9_	300	-	-	-	-	-
4c	C_7_H_15_	-	-	-	-	-	-
4d	CH_2_C_6_H_5_	-	-	-	-	-	-
4e	CH_2_C_6_H_4_(2-F)	100	-	100	100	-	-
4f	CH_2_C_6_H_4_(3-F)	100	-	100	100	-	-
4g	CH_2_C_6_H_4_(4-F)	100	30	30	30	300	300
4h	CH_2_C_6_H_4_(2-Cl)	100	100	-	300	-	-
4i	CH_2_C_6_H_4_(3-Cl)	100	100	-	-	-	-
4j	CH_2_C_6_H_4_(4-Cl)	300	-	-	-	-	-
4k	CH_2_C_6_H_3_(2,4-Cl2)	300	-	-	-	-	-
4l	CH_2_C_6_H_4_(4-CH3)	300	300	-	300	-	-
4m	CH_2_C_6_H_4_(4-OCH3)	-	-	-	-	-	-

^a^ Number of animal used = 3–5, Doses of 15, 30, 100 and 300 mg/kg were administered. The figure in the table indicates the minimum dose whereby bioactivity was demonstrated in half or more of the mice. The animals were examined at 0.5 and 4 h after injection was administered. A dash indicates the absence of anticonvulsant activity and neurotoxicity at the maximum dose administered (300 mg/kg); ^b^ Maximal electroshock test; ^c^ Subcutaneous pentylenetetrazole test; ^d^ Neurotoxicity screening (rotarod test).

**Table 2:** Quantitative anticonvulsant date of **4g** (anti-MES and anti-PTZ) in mice administered intraperitoneally.

Compounds	ED_50_^a^		TD_50_^b^	PI ^c^	
	MES	scPTZ		MES	scPTZ
**4g**	23.7 (21.5–26.0)	18.9 (17.2–20.8)	284.0 (258.2–312.4)	12.0	15.0
Carbamazepine	9.8 (8.9–10.8)	>100	44.0 (40.2–48.1)	4.5	<0.44
Valproate	264 (247–338)	149 (123–177)	418 (369–450)	1.6	2.8

Ten animals were used for each group; polyethylene glycol 400 (0.1 mL, i.p.) was used as solvent. ^a^ ED_50_—median effective dose required to assure anticonvulsant protection in 50% animals; ^b^ TD_50_—median toxic dose eliciting minimal neurological toxicity in 50% animals; ^c^ PI—protective index (TD_50_/ED_50_).

**Table 3:** Effect of compound **4g** on 3-mercaptopropionic-acid-induced seizures in mice.

Compound	Doses (mg/kg)	Test Time (h)	Clonic Seizures (%)	Tonic Seizures (%)	Lethality (%)
DMSO	-	0.5	100	100	60
Carbamazepine	30	0.5	100	0 ***	0 **
**4g**	30	0.5	100	20 **	30 *

Results are expressed as a percentage of animals that showed clonic and tonic convulsions and death among all animals tested. Ten mice were included in each group. Significance determined with Fisher’s exact test. * *p* < 0.05, ** *p* < 0.01, *** *p* < 0.001 when vs. control group (3-MP, 60 mg/kg).

**Table 4:** Effect of compound **4g** on Bicuculline-induced seizures in mice.

Compound	Doses (mg/kg)	Test Time (h)	Clonic Seizures (%)	Tonic Seizures (%)	Lethality (%)
DMSO	-	0.5	100	100	100
Carbamazepine	30	0.5	100	0 ***	20 **
**4g**	30	0.5	100	0 ***	30 **

Results are expressed as a percentage of animals that showing clonic and tonic convulsions and death among all animals tested. The number of animals tested in each group was ten. Significance determined with Fisher’s exact test. ** *p* < 0.01, *** *p* < 0.001 when vs. control group (BIC, 5.4 mg/kg).
